# EDI and open access: How JACMP is the future of ethical publishing—A tale in two parts

**DOI:** 10.1002/acm2.13818

**Published:** 2022-11-04

**Authors:** Samantha G. Hedrick, Susan Richardson

**Affiliations:** ^1^ Thompson Proton Center Knoxville Tennessee USA; ^2^ Department of Radiation Oncology Swedish Medical Center – Tumor Institute Seattle Washington USA

At its core, scientific publishing in healthcare is meant to help society. A scientist is researching and presenting data that will influence decisions for patient care. Some data is meant to help patients in the clinic now, some data might help patients 5 or 10 years in the future. Secondarily, publishing can help the scientist, allowing the author to advance in their career and continue to research and publish. I believe it is safe to say that each Medical Physicist wants to see our scientific community improve and advances made in care for our patients. A crucial step in this improvement is ensuring that all the best minds are working toward this goal. To get the best minds in our community, we must integrate equity, diversity, and inclusion (EDI) into our scientific publishing process.

EDI is a convenient acronym and easy to view as a single idea, but each component should be considered individually. Equity, as defined by the Merriam–Webster, is “justice, according to natural law or right.” It is often used to describe what is “just” and “fair.” It can also often be confused with equality, which is “the quality or state of being equal.” Essentially, equity is fairness and equality is sameness. Equity is often used to describe the resources provided to an individual. Equitable resources could mean providing different resources for different people, depending on their needs. Equity can be a tricky concept, because it can be argued that it is not fair to provide different resources to one group and not another. This also raises the question of merit. It can be argued that resources should be provided to those that have earned them, based on their quality, not on their need. Diversity is “the condition of having or being composed of differing elements.” Regarding scientific publishing, it is easy to think of diversity only in terms of race and gender. But there are a multitude of ways in which people can be different. Diversity should be considered in the data we are collecting, the authors writing the manuscripts, and the editors and reviewers who are reviewing the manuscripts. The definition of inclusion is straightforward, “the act of including: the state of being included.” However, the integration of inclusion can be less straightforward. Often, inclusion, or the lack thereof, is presented in the resources we develop for the public, the language used to invite editors and authors, and the reviews delivered to the authors.

EDI is a hot‐button topic and can be polarizing in our community, but it is important to evaluate and discuss. The entire scientific publishing process, from the data we collect to the reviewers reading the papers, has some room for improvement that will ultimately enhance our patients’ lives and our Medical Physics community. The first step in the publishing process, and our EDI evaluation, is collecting and assessing the data.

A valuable resource for data in radiation oncology is clinical trials. Over the years, clinical trials have improved to protect patients, incorporating the four principles of medical ethics, including autonomy, beneficence, non‐maleficence, and justice. These principles are essential for developing clinical trials and protecting the patients on whom research is being conducted. Part of ensuring beneficence, non‐maleficence, and justice is considering the diversity of the data being collected. A homogeneous group of research patients could mean that other groups are missing out on the opportunity to participate in the trial and benefit from the testing. It could also mean that the outcome of the data is not applicable to a diverse group of patients, and these other groups are not able to benefit from the treatment. A recent study investigating the exclusion of females in clinical trials found that females were underrepresented compared with their proportion of disease population in cardiovascular trials (41.9% vs. 49%), psychiatry clinical trials (60% vs. 42%), and cancer trials (51% vs. 41%).^1^ Without female inclusion in clinical trials, toxicity‐ and treatment‐related sex‐based differences remain a mystery. For instance, it has been demonstrated that women are more susceptible to toxicity from various chemotherapy regimens.^2^ In another study, Clark et al. evaluated racial diversity in clinical trials and found that, while the US population in 2019 was 16% Hispanic and 12% African American, the representation of Hispanics and African Americans in clinical trials was only 1% and 5%, respectively.^3^ The paper cites barriers faced by racial and ethnic minorities in clinical trial research, such as “mistrust, lack of comfort with the clinical trial process, lack of information about clinical trials, time and resource constraints associated with participation, and lack of awareness about the existence and importance of clinical trials.” They found that improved communication is key to overcoming these barriers. Additionally, providing equitable resources, such as “transportation, flexible hours for patients, appropriate compensation, and mobile technology support such as an app for patients and cell phones for those who do not have one,” can also improve minority participation in clinical trials. They define the roles and responsibilities of key stakeholders in providing these resources and overcoming barriers.

Diversity should also be considered in the authors writing the publications. A study in 2022 from Kozlowski et al. looked at the racial and gender diversity of authors in scientific publications. From more than 5 million articles, they evaluated 1.6 million distinct US first authors and inferred race and gender. They found that, in health science, Latinx, Black, and White women are overrepresented as authors. However, in physics and mathematics, they are underrepresented.^4^ Diversity in authors goes beyond race and gender, and other factors should also be considered, such as geographical location, clinic type (academic vs. private, small clinic vs. large) education and experience, socioeconomic status, access to resources, monetary and management support, physical abilities and disabilities, and many others. This is another step in which equitable resources can and should be provided to increase the diversity of authors publishing in our community and the JACMP, specifically.

The value of incorporating EDI in the scientific publishing process has been studied in several different ways. AlShebli et al., in 2018, looked at the impact of ethnic diversity in scientific collaboration.^5^ By evaluating citations within 5 years of publication, they found that ethnic diversity is strongly correlated with impact. A paper written by an ethnically diverse group of authors has a higher impact factor than one written by a homogenous group of authors. Similarly, a scientist surrounded by a diverse group of collaborators has a higher impact factor than an ethnically similar group of collaborators.

**FIGURE 1:**
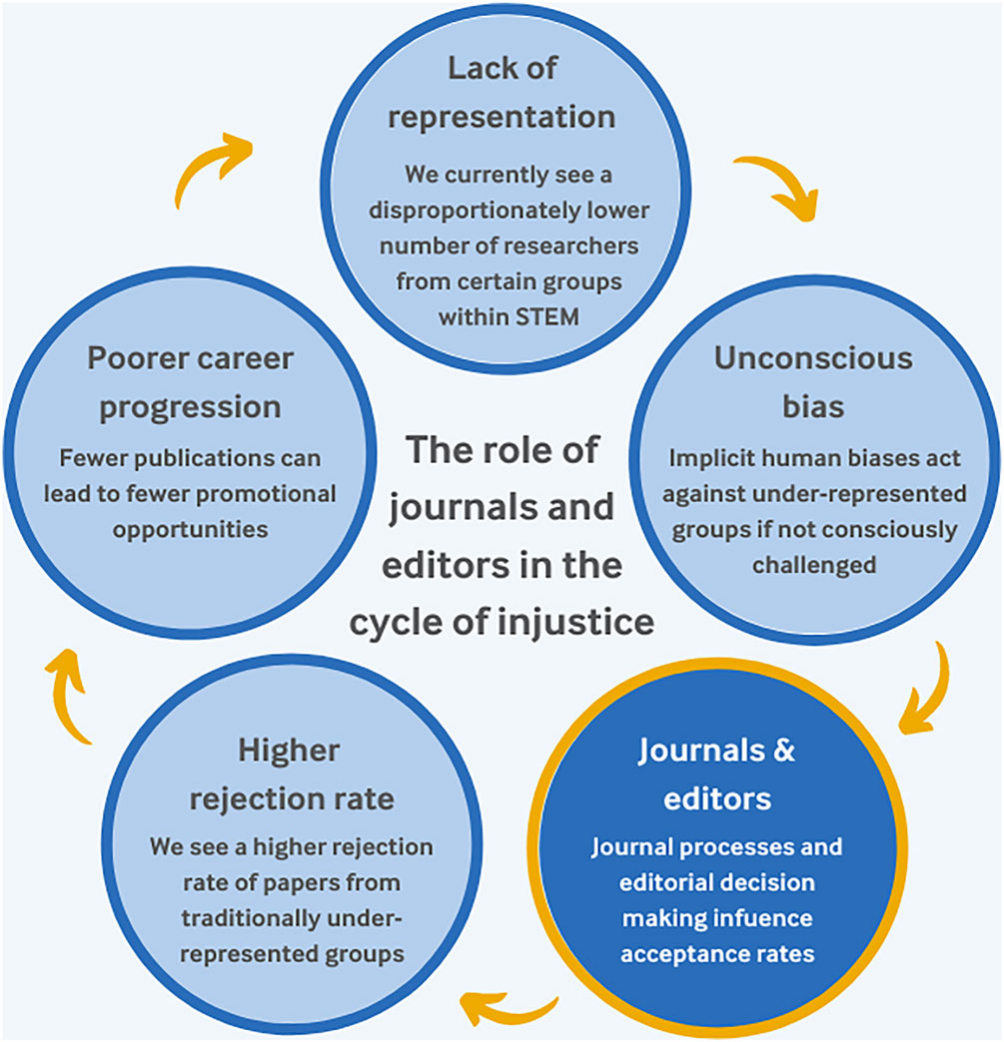
“The cycle of injustice,” as outlined by the BMJ^9^

Several groups have evaluated the status of EDI in scientific publishing and found that there is room for improvement. Salazar et al., in 2021, found that most editors of top‐cited journals are straight White men.^6^ Also in 2021, Chatterjee et al. found that research conducted by women received fewer citations.^7^ In 2013, Hopkins et al. found that there is a disproportionately higher rejection rate for authors from underrepresented groups.^8^ In response to studies such as these, the BMJ developed what they call the “Cycle of Injustice,” shown in Figure 1, and outlined the role of journals and editors in that cycle.^9^ They explain that journals and editors influence acceptance rates for papers, ultimately influencing the rejection rates. If we are seeing a higher rejection rate for traditionally underrepresented groups, then these groups of people will likely have poorer career progression due to fewer publications. These groups will continue to see a lack of representation in our community, which will further enforce the unconscious bias that already exists against these underrepresented groups.

There are several types of unconscious bias that could exist in the publishing process. Bias is a prejudice in favor of or against one thing, person, or group that is usually considered to be unfair. Unconscious bias, also known as implicit bias, is a bias that is formed outside one's own conscious awareness. Everyone experiences unconscious bias, and it is typically based on social stereotypes toward peoples of various races, ethnic groups, gender identities, sexual orientations, physical abilities, education, and more. One example of unconscious bias is affinity bias, which is gravitating toward people similar to ourselves. In scientific publishing, it might mean a reviewer is more likely to positively review an article from an author with similar training, education, or background. Another example is confirmation bias, which is searching for information to confirm one's own beliefs, rather than objectively evaluating the data or information.^10^ Another common bias, particularly in publishing, is linguistic discrimination. It can be challenging to evaluate the science of the manuscript, separate from the spelling, grammar, and syntax. These biases are difficult to overcome, most are not aware they are occurring. Organizations within publishing are pursuing concepts to begin to address these concerns.

In 2020, the Royal Society of Chemistry (RSC) hosted a workshop, bringing together 53 publishing organizations to set new standards for diversity and inclusivity within scholarly publishing. From this workshop came the “Joint commitment for action on inclusion and diversity in publishing.”^11^ The joint commitment promised to “understand our research community, reflect the diversity of our community, share success to achieve impact, and set minimum standards on which to build.” When the joint commitment was launched in 2020, 12 publishers signed on. As of the writing of this editorial, over 50 publishers have joined this commitment, including Wiley that publishes the JACMP. The RSC outlined the importance of EDI in scholarly publishing, stating that inclusion and diversity ensures “entry of new researchers and opportunities for researchers of all backgrounds to advance and excel throughout their careers, a wider range of topics and research questions will be pursued, rigorous, reproducible and higher‐quality research studies; and equitable and widespread impact of research outcomes to benefit all of society. Societal challenges of our time make it necessary for us to harness the inclusive contribution of diverse researchers to deliver equitable impact.”

One of the tasks of the joint commitment was to develop standardized questions to collect diversity data. In the publication reviewing process, it is common practice for a blind or double‐blind review, which means the reviewers cannot see the author demographics and the authors cannot see the reviewer demographics. This blind review is an effort to remove bias that could exist based on an author's name and affiliation. Because of this process, it seems natural to ignore gender, race, and ethnicity data for authors, reviewers, and editors because it seemingly helps eliminate bias. However, by not collecting this data, the community is unaware of the diversity, or lack thereof, of the authors, reviewers, and editors. The first task of the joint commitment is to understand our publishing community, so the data must be collected. The standardized questions are as follows^12^:

**Table:** 

**Gender identity**:
With which gender do you most identify? Please select one option:
Woman
Man
Nonbinary or gender diverse
Prefer not to disclose
**Race and ethnicity**:
What are your ethnic origins or ancestry? Please select ALL the geographic areas from which your family's ancestors first originated:
Western Europe (e.g., Greece, Sweden, and United Kingdom)
Eastern Europe (e.g., Hungary, Poland, and Russia)
North Africa (e.g., Egypt, Morocco, and Sudan)
Sub‐Saharan Africa (e.g., Kenya, Nigeria, and South Africa)
West Asia/Middle East (e.g., Iran, Israel, and Saudi Arabia,)
South and Southeast Asia (e.g., India, Indonesia, and Singapore)
East and Central Asia (e.g., China, Japan, and Uzbekistan)
Pacific/Oceania (e.g., Australia, Papua New Guinea, and Fiji)
North America (Canada and United States)
Central America and Caribbean (e.g., Jamaica, Mexico, and Panama)
South America (e.g., Brazil, Chile, and Colombia)
Self‐describe* [open text box]
Prefer not to disclose
How would you identify yourself in terms of race? Please select ALL the groups that apply to you:
Asian or Pacific Islander
Black
Hispanic or Latino/a/x
Indigenous (e.g., North American Indian Navajo, South American Indian Quechua, Aboriginal or Torres Strait Islander)
Middle Eastern or North African
White
Self‐describe* [open text box]
Prefer not to disclose

* where system functionality does not permit the collection of free‐text responses, the use of “other” is an acceptable alternative to “self‐describe.” Some publishers may also choose not to include a “self‐describe” or “other” option.

Currently, Wiley does not collect diversity data from anyone within the publishing process, including readers, authors, reviewers, or editors. There is a large amount of data available to evaluate readership and authorship, specifically what country is downloading and submitting data. By collecting diversity data from the readers, authors, reviewers, and editors, publishers can understand the diversity of the Medical Physics community and begin to make changes and ensure that diversity is reflected in every step of the publishing process. At the time of publication, 227 countries have downloaded at least one article from the JACMP over the last 5 years. Compare that to the number of countries with authors for the JACMP, which is only 51 countries. A total of 25 authors in 2022 had unknown country origin. Authors from the United States account for ∼50% of JACMP authors for the last 5 years, but only account for 30% of the downloads.

The next task of the joint commitment was to develop minimum standards on which publishers can build. The purpose of these minimum standards is to enable publication leadership to evaluate their performance and progress on inclusion and diversity and take specific actions to improve. The minimum standards are as follows:

1. Ensure that inclusion and diversity are integrated into publishing activities and strategic planning.
2. Work to understand the demographic diversity of authors, editorial decision makers and reviewers, such as gender, geography, and ethnicity data.
3. Acknowledge the barriers within publishing which authors, editorial decision makers, and reviewers from underrepresented communities experience and take actions to address them.
4. Define and communicate the specific responsibilities authors, editorial decision makers, reviewers and staff members have toward inclusion and diversity.
5. Review and revise as appropriate the appointment process for editors and editorial boards to capture the widest talent pool possible.
6. Publicly report on progress on inclusion and diversity in scholarly publishing at least once a year.

Each member of the joint commitment has agreed to abide by these minimum standards, but there are no defined methods for the process. Publishers have posted statements on their websites, detailing certain actions they will take to meet these minimum standards. One such action is providing gender equality. An example of this effort is eliminating “manels,” which are panels made entirely of men. As of 2021, about 75% of the AAPM self‐reported as men, 24% were women, and 1% preferred not to respond.^13^ The panels, committees, and boards representing the AAPM community should reflect the diversity of our membership. Ideally, a panel should be at least 25% women. Looking at racial diversity, the AAPM Climate Survey found that only 36% of Medical Physicists identifying as Asian or Asian‐American felt there was opportunity in AAPM leadership, compared to 55% of White US/Canadian origin.^13^ Another example is permitting authors to change their name post‐publication, in the case of a gender identity, religion, or relationship status change. This has historically been permitted; however, it required the publication of a correction notice and notification to the coauthors. Publishers are now removing the notification requirements, so an author can change their name with more anonymity. The double‐blind review has been used for many years with many publishers. It prevents reviewers from seeing the names and affiliations of authors and prevents the authors from seeing the names and affiliations of reviewers. The concept, ideally, removes the bias that could exist when a reviewer makes assumptions about a manuscript based on the authors name and affiliation.

Another action to improve diversity in publishing, beyond gender, is encouraging participation from underrepresented groups in both authorship and reviewers. One example of an underrepresented group is those from the global south, including Latin America, Africa, developing countries in Asia, and Oceania. Researchers in this region could face socioeconomic and systemic barriers, limiting their access or opportunities within publishing. For many of these regions, English is a second language, so there are barriers to publishing in major scientific publications that produce manuscripts only in English. There is a push for publications to provide equitable resources to these authors, so they have the same opportunities for authorship as those in the global north. Publishers can provide author services for those who use English as a second language, helping these authors follow English grammar and syntax conventions, develop a clear research story, interpret formatting instructions from the website, and decipher feedback from reviewers.

Moving beyond the authors, it is important to consider EDI in the review process, including the editors, reviewers, and reviewing process. The Joint Commitment has tasked the leadership of publications, including the editorial board, to consider EDI in their decision‐making processes. One example is actively seeking diversity when developing editorials, commentary, blogs, and specials issues. Another example is updating the public‐facing resources available about the publication, ensuring that websites and other resources include inclusive language and describe the efforts of the journal to improve EDI. As noted earlier, by Salazar, there is a lack of diversity in editorial boards of journals, so leadership should also seek to diversify these boards. Some methods to improve this diversity includes ensuring inclusive language in the invitation to join the board, asking current board members to nominate colleagues from underrepresented groups, increasing the number of members on the board to gain diversity, and offering a fellowship to train potential board members. Publishers have also been tasked with developing reporting guidelines for diversity in human research. As mentioned earlier, there are underrepresented groups within clinical trials and other human research, leading to unequitable distribution of treatments and outcomes. If publishers require diversity data to be reported in the manuscript, it will bring awareness to the problem.

In addition to the editors, the reviewer pool should also be evaluated for diversity and inclusivity. Similar to the editorial board, the reviewer invitation should be evaluated for inclusive language. Additionally, public‐facing resources should be updated to encourage authors to recommend reviewers from underrepresented backgrounds. As mentioned before, an important component of ensuring a diverse reviewer pool is collecting demographic data, which is not commonly performed. It is also important for journals to provide resources to train editors and reviewers, particularly in unconscious bias.^14^ The double‐blind review eliminates the unconscious bias associated with an author's name and affiliation, but there are other biases that could occur when reviewing a manuscript. The confirmation bias can influence the assessment of research quality if the reviewer thinks the data supports their own beliefs. There are many studies evaluating the impact of bias and the effectiveness of peer review.^15^, ^16^, ^17^

Finally, it is important to think about the readers of the journals, evaluating the reach and impact of the journal. There are barriers that could prevent equal access to these scientific publications, such as the cost of a subscription. This will be covered in a subsequent letter to the editor and remains an important component of enhancing EDI within the publication system.

The Wiley and the JACMP are committed to advancing equity, diversity, and inclusivity within the journal and the entire publishing process. Some current efforts include the signing of the Joint Commitment for Action on Inclusion and Diversity in Publishing, utilizing double blind review, and providing open access to all JACMP articles. Future endeavors, aligning with the Joint Commitment, will likely include collecting diversity data from authors and reviewers, pushing the JACMP to the forefront of ethical publishing. We cannot do our best work without including the best minds. As Mother Teresa said, “I can do things you cannot, you can do things I cannot; together we can do great things.”
